# Supplementary material for: Association between oral microbiome and seven types of cancers in East Asian population: a two-sample Mendelian randomization analysis
Source: Front Mol Biosci. 2023 Nov 21;10:1327893. doi: 10.3389/fmolb.2023.1327893 (PMC10702768; doi:10.3389/fmolb.2023.1327893)
Supplement: Supplementary file 1 [file DataSheet1.ZIP › Supplementary Materials/MR plots of saliva/saliva═╝/ovarian cancer/pheno.2523_to_ovarian cancer_leave_one_out.pdf]

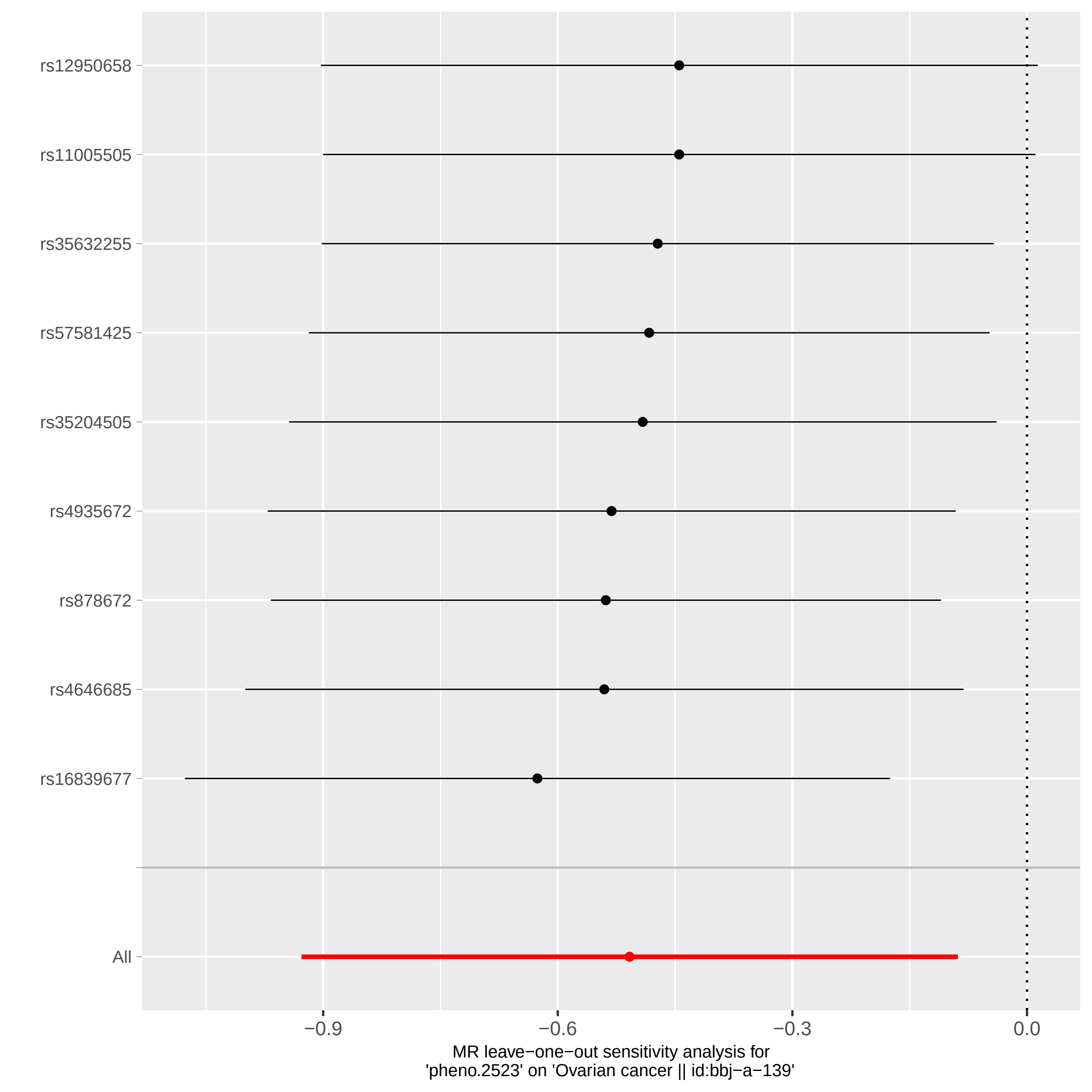
rs12950658
rs11005505
rs35632255
rs57581425
rs35204505
rs4935672
rs878672
rs4646685
rs16839677
All
−0.9
−0.6
−0.3
0.0
MR leave−one−out sensitivity analysis for
'pheno.2523' on 'Ovarian cancer || id:bbj−a−139'
